# Effects of histidine protonation and rotameric states on virtual screening of *M. tuberculosis* RmlC

**DOI:** 10.1007/s10822-013-9643-9

**Published:** 2013-04-12

**Authors:** Meekyum Olivia Kim, Sara E. Nichols, Yi Wang, J. Andrew McCammon

**Affiliations:** 1 Department of Chemistry and Biochemistry, University of California San Diego, La Jolla, CA 92093 USA; 2 Department of Pharmacology, University of California San Diego, La Jolla, CA 92093 USA; 3 Center for Theoretical Biological Physics, University of California San Diego, La Jolla, CA 92093 USA; 4 Howard Hughes Medical Institute, University of California San Diego, La Jolla, CA 92093 USA; 5 Present Address: Department of Physics, The Chinese University of Hong Kong, Shatin, NT Hong Kong

**Keywords:** Docking, Drug design, Histidine, Protonation state, Rotameric state, Virtual screening

## Abstract

While it is well established that protonation and tautomeric states of ligands can significantly affect the results of virtual screening, such effects of ionizable residues of protein receptors are less well understood. In this study, we focus on histidine protonation and rotameric states and their impact on virtual screening of *Mycobacterium tuberculosis* enzyme RmlC. Depending on the net charge and the location of proton(s), a histidine can adopt three states: HIP (+1 charged, both δ- and ε-nitrogens protonated), HID (neutral, δ-nitrogen protonated), and HIE (neutral, ε-nitrogen protonated). Due to common ambiguities in X-ray crystal structures, a histidine may also be resolved as three additional states with its imidazole ring flipped. Here, we systematically investigate the predictive power of 36 receptor models with different protonation and rotameric states of two histidines in the RmlC active site by using results from a previous high-throughput screening. By measuring enrichment factors and area under the receiver operating characteristic curves, we show that virtual screening results vary depending on hydrogen bonding networks provided by the histidines, even in the cases where the ligand does not obviously interact with the side chain. Our results also suggest that, even with the help of widely used pKa prediction software, assigning histidine protonation and rotameric states for virtual screening can still be challenging and requires further examination and systematic characterization of the receptor-ligand complex.

**Electronic supplementary material:**

The online version of this article (doi:10.1007/s10822-013-9643-9) contains supplementary material, which is available to authorized users.

## Introduction

The effect of ligand protonation and tautomeric states on virtual screening (VS) has been the subject of extensive research [1–4]. It is well known that different protonated forms or tautomers of a ligand may have significantly different rankings in VS [1, 2]. Unlike ligand molecules, for which multiple tautomers and protonated forms can be included in a VS study, the ionizable residues of protein receptors are assigned a single state prior to the screening. For instance, in the standard protonation model, all Asp, Glu, and His residues are deprotonated while all Arg and Lys residues are protonated. Various algorithms, such as PROPKA [5–8], H++ [9–11], and MCCE [12–14], have been developed to improve the quality of the proton assignment. However, few studies have investigated the effect of such assignment of the titratable residues of protein receptors on VS results [15]. In this study, we focus on the impact of histidine protonation and rotameric states on VS by systematically analyzing a screen using results from a previous high-throughput screening (HTS) of the enzyme RmlC (dTDP-6-deoxy-d-*xylo*-4-hexulose 3′,5′-epimerase) of *Mycobacterium tuberculosis* (*Mtb*) [16].

**Fig. 1:**
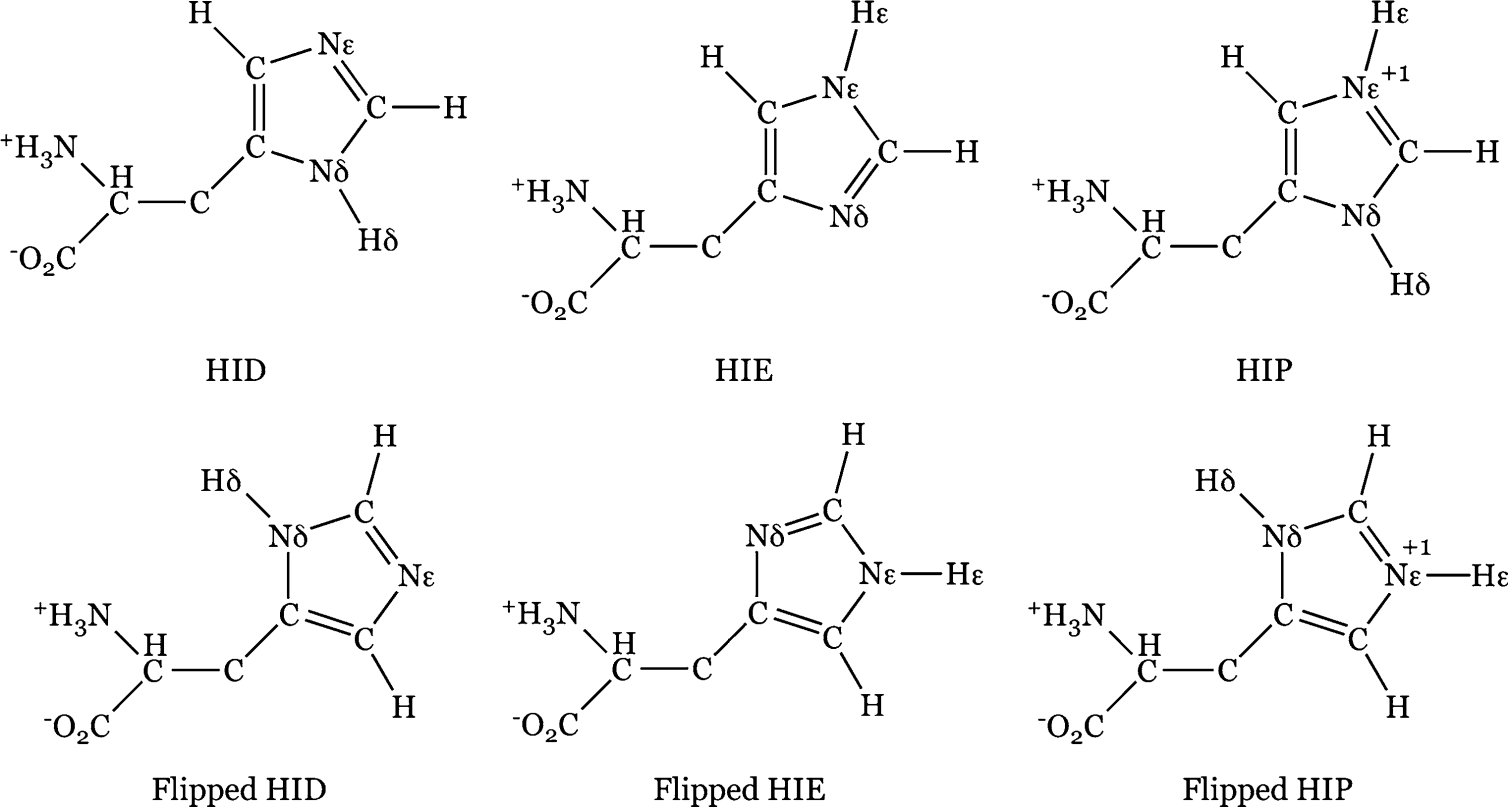
Six possible protonation and rotameric states of a histidine. Formal charges on nitrogen in HIP states are marked

Histidines participate in a large number of important biochemical reactions. Their roles as catalytic residues in the enzymatic active site [17], as proton shufflers in proton transfer reactions [18–20], as coordinating ligands in metalloproteins and hemoglobin [21, 22] render histidines essential for proper function of a cell. The side chain of a histidine has a pKa around 6.0, which is close to the physiological pH [23]. Depending on the pH of its environment, a histidine readily switches between the doubly protonated cationic form and the neutral state (Fig. 1): At low pH, both δ-nitrogen and ε-nitrogen of the imidazole ring are protonated and the amino acid has a +1 charge (HIP). At high pH, the histidine is neutral with either δ-nitrogen (HID) or ε-nitrogen (HIE) protonated. Apart from the above three states, positions of carbon and nitrogen atoms in the imidazole ring may be switched due to common ambiguities in X-ray crystal structures [24]. As a result, a histidine can adopt three additional rotameric states, namely, flipped HIP, flipped HID, or flipped HIE (see Fig. 1) [25]. In this work, we set out to evaluate the impact of all six protonation and rotameric states of a histidine on the virtual screening results.

**Fig. 2:**
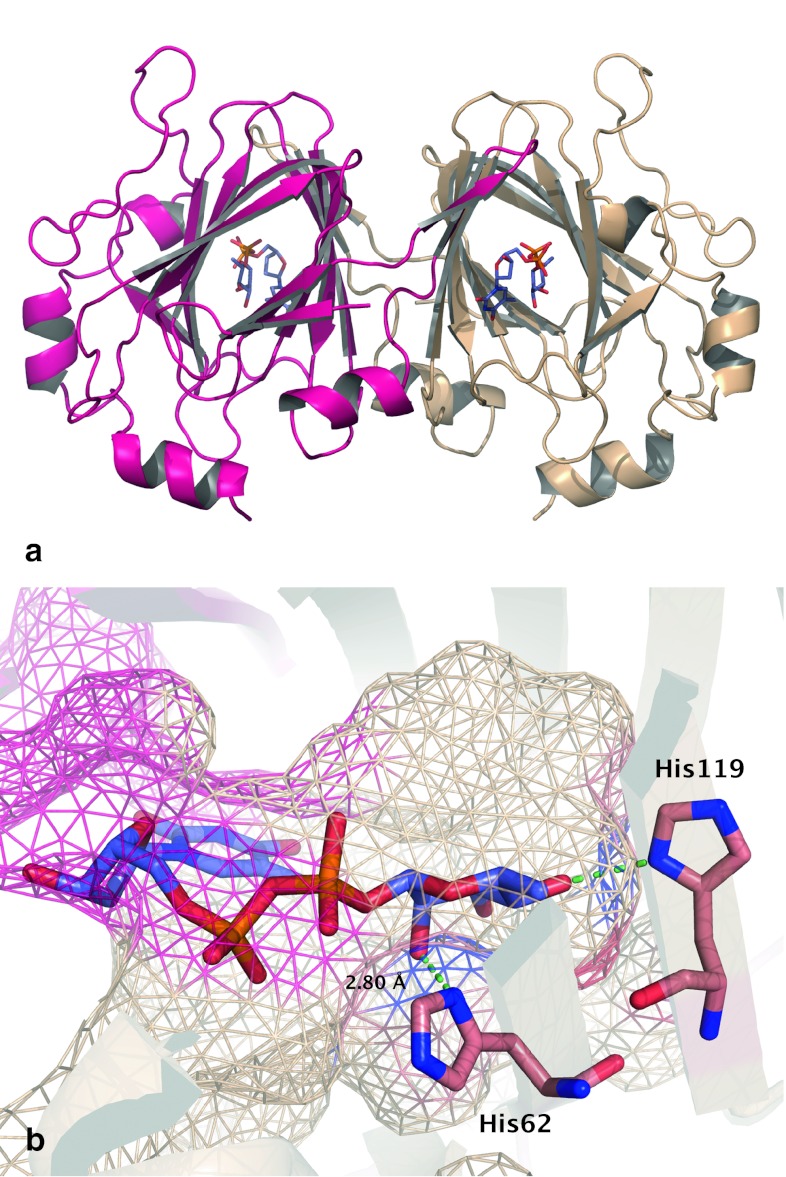
**a** RmlC homodimer in complex with co-crystalized 2′-deoxy-thymidine-β-l-rhamnose (TRH) (PDB ID: 2IXC). The two monomers are *colored* in *pink* and *beige*, respectively. **b** Close view of the co-crystal ligand TRH, with His62 and His119 *highlighted*. TRH is *colored* with carbon in *violet*, nitrogen in *blue*, oxygen in *red*, and phosphorus in *orange*. Protein residues are *colored* with carbon in *salmon*, while other atoms are following the *same* *coloring scheme* as TRH. The binding surface of receptor is represented as *wire frame*. Hydrogen bonds are shown with *dashed green lines*

*Mycobacterium tuberculosis* is the primary causative pathogen of the lethal, contagious disease tuberculosis (TB). It has a three-layered cell wall composed of peptidoglycan, arabionogalactan, and mycolic acids [26]. This highly impermeable cellular envelope provides natural resistance against a large variety of antibiotics, which renders the inhibition of the cell wall biosynthesis a promising target for anti-TB drug discovery [16, 26]. The enzyme RmlC participates in the synthesis of an indispensible linker molecule dTDP-l-rhamnose (TDP-Rha), connecting the peptidoglycan and arabinogalactan layer in the *Mtb* cell wall [6, 16]. Based on the crystal structure of the *Mtb* RmlC in complex with TDP-Rha (Fig. 2a), it has been suggested that the enzyme uses a histidine (His62) as a key catalytic site that pairs with Tyr132 in an acid–base couple for proton transfer [27]. Apart from His62, the active site contains another histidine (His119) involved in the interaction with TDP-Rha.

As a part of a drug discovery campaign against TB, 201,368 compounds were screened in a previous HTS against RmlC, revealing a series of hits with the best half inhibitory concentration (IC_50_) of 0.12 μM at pH 7.4 [16]. Based on these results, we constructed a library of 2,010 compounds, including 2,000 decoys and ten actives. The library was screened against 36 receptor models with different protonation and rotameric states of His62 and His119 of RmlC. Through enrichment factors (EF), receiver operating characteristic (ROC) curves, and area under the curve (AUC) metrics, we systematically evaluated the relative VS performance of various protonated receptor models. In the remainder of the text, we will discuss these analyses in detail and examine pKa predictions for the two histidines made by commonly used software packages.

## Methods

### Crystal structure and initial preparation

The crystal structure of RmlC in complex with the product analog 2′-deoxy-thymidine-β-l-rhamnose (TRH) was obtained from Protein Data Bank (PDB ID: 2IXC [27]). One dimer containing chains A and B, each in complex with a TRH molecule, was submitted to the Protein Preparation Wizard of Schrodinger Suite 2011 [28]. Missing hydrogen in the crystal structure were added while water and TRH molecules were removed, followed by a brief optimization of hydrogen positions at pH = 7.0. Receptor models with 36 different protonation and rotameric states of His62 and His119 in chain A were then generated and energy refined with the OPLS2005 force field. Two other titratable residues in the active site, Lys72 and Asp83, were kept charged. Subsequent virtual screening was performed on the active site of chain A. See Online Resource 1 for a schematic description of the hydrogen and nitrogen of His62 and His119 acting as a hydrogen bond donor or acceptor in each receptor model.

### Receptor grid generation

A set of 36 receptor models based on different protonation states of His62 and His119 were generated using Glide 5.7.111 in Schrodinger Suite 2011 [29]. The grid center was set to where the center-of-mass of the TRH molecule in chain A had been before removal. The sizes of the inner and outer grid boxes were set to 10 and 20 Å in each direction, respectively. The models were assigned unique numbers from 1 to 36 as listed in Table 1.

**Table 1:** AUC values of 36 receptor models with different protonation and rotameric states

Receptor model	His62	His119	AUC	EF^1%^	EF^10%^
1	HIE	HIE	0.942	60	8
2	HIE	Flipped HIE	0.992	60	10
3	HIE	HID	0.868	30	5
4	HIE	Flipped HID	0.961	50	9
5	HIE	HIP	0.875	30	6
6	HIE	Flipped HIP	0.996	80	10
7	Flipped HIE	HIE	0.963	70	9
8	Flipped HIE	Flipped HIE	0.945	30	7
9	Flipped HIE	HID	0.991	60	10
10	Flipped HIE	Flipped HID	0.938	50	6
11	Flipped HIE	HIP	0.918	0	8
12	Flipped HIE	Flipped HIP	0.963	60	8
13	HID	HIE	0.989	30	10
14	HID	Flipped HIE	0.991	40	10
15	HID	HID	0.989	30	10
16	HID	Flipped HID	0.990	20	10
17	HID	HIP	0.916	0	8
18	HID	Flipped HIP	0.991	20	10
19	Flipped HID	HIE	0.992	80	10
20	Flipped HID	Flipped HIE	0.957	40	8
21	Flipped HID	HID	0.969	50	9
22	Flipped HID	Flipped HID	0.987	50	10
23	Flipped HID	HIP	0.981	40	10
24	Flipped HID	Flipped HIP	0.988	60	10
25	HIP	HIE	0.971	40	8
26	HIP	Flipped HIE	0.991	30	10
27	HIP	HID	0.982	0	10
28	HIP	Flipped HID	0.933	40	9
29	HIP	HIP	0.869	0	7
30	HIP	Flipped HIP	0.936	30	9
31	Flipped HIP	HIE	0.945	30	9
32	Flipped HIP	Flipped HIE	0.933	10	6
33	Flipped HIP	HID	0.964	40	8
34	Flipped HIP	Flipped HID	0.917	0	6
35	Flipped HIP	HIP	0.950	0	8
36	Flipped HIP	Flipped HIP	0.969	20	9

### Ligand preparation

A library containing 2,000 inactive and ten active compounds was generated from the previous HTS result of the NIH Molecular Libraries Small Molecule Repository (BioFocus DPI) [16]. First, the entire library of 201,386 compounds used in the HTS was obtained from PubChem, with BioAssay IDs 1532, 1533, 1695, and 1696 (including primary screening results and dose–response assays) [16]. Ten verified actives were selected from BioAssay 1696 and 2,000 inactive compounds were randomly selected from the remaining compounds. The final library of 2,010 ligands was then subjected to LigPrep of Schrodinger Suite 2011 [30] with OPLS2005 force field. The ligands were ionized at pH = 7.0 ± 2.0 using Epik [31, 32] and tautomers and stereoisomers were generated for the inactives, resulting in a library of total 3,934 compounds. The Canvas tool of Schrodinger Suite 2012 [33–35] was used to compare the similarity of the actives and decoys. Tanimoto coefficients of the compounds in the library to each of the actives were calculated based on the molecular binary fingerprints, as described in Online Resource 2. The co-crystal ligand TRH was also prepared in the same way and docked to all models for initial assessment of pose prediction.

### Docking

After experimenting with both the Glide SP and XP docking modes [29, 36–38] we found that XP outperformed SP in ranking the actives over decoys (data not shown). The different performance of SP and XP mainly stems from differences in their scoring functions. The hydrophobic enclosure term in the XP algorithm may be particularly suitable for our study, given the strong hydrophobic interactions between many active compounds and the binding site [38]. Hence, in the remainder of the study, we used the Glide XP mode to perform docking on 36 receptor models described above.

### Predictive performance analysis

We analyzed the predictive performance of VS using 36 receptor models described above by calculating enrichment factors (EF), receiver operating characteristic (ROC) curves, and areas under the curve (AUC). The statistical significance of the AUC values of different receptor models was evaluated with a *p* test with 95 % confidence limit.

The EF is a widely used metric to evaluate the efficiency of VS [39]. The value of EF^x%^ indicates how much more likely an active compound is ranked in the top x% of a VS result compared with a random selection, i.e., how many times the database is enriched. Specifically, EF is calculated as Eq. 1:

1 $$ {\text{EF}}^{{{\text{x}}\% }} = \frac{{{\text{N}}_{\text{experimental}}^{{{\text{x}}\% }} }}{{{\text{N}}_{\text{active }} \times {\text{x}}\% }} $$

where N_experimental_^x%^ is the number of experimentally verified actives in the top x% of the database and N_active_ is total number of actives in the database [39]. In this study, EF^1%^ and EF^10%^ were calculated from the top 1 and 10 % of the VS result, respectively.

To investigate the docking performance in a threshold-independent manner, the AUC value was calculated from the ROC curve. The ROC curve allows a straightforward visualization of the performance of VS in ranking the actives higher over decoys [40]. In our study, we have a list of experimentally verified actives, or positives, and decoys, or negatives. These positives and negatives are further categorized into true or false according to their rank above or below, respectively, a certain threshold of the VS result, i.e., the actives ranked above a chosen threshold becomes true positive (TP). To generate the ROC curve, the true positive rate (TPR) and false positive rate (FPR) are calculated as Eqs. 2 and 3:

2 $$ {\text{TPR}} = {\text{TP}}/({\text{TP}} + {\text{FN}}) $$

3 $$ {\text{FPR}} = {\text{FP}}/({\text{TN}} + {\text{FP}}) $$

In the ROC curve, the TPR is plotted as a function of the FPR. The AUC was then calculated to compare the performance of different receptor models quantitatively [23]. An AUC of 0.5 corresponds to a random selection of the ligand by a receptor.

To evaluate the statistical significance of the AUC values of different receptor models, we performed the two-sided *p* test at the 95 % level. A two-sided *p* value of less than 0.05 (corresponding to 5 %) rejects the null hypothesis that the AUC values of a pair of receptors are statistically identical and accepts the alternative hypothesis that their difference is statistically meaningful. Hence the pair of receptors with statistically different AUC values is differentiated by their abilities to rank the actives and decoys. The two-sided *p* values were calculated following Craig et al. and references therein [41, 42], which is described below briefly. As in Eq. 4, the AUC is first calculated as the mean TPR of decoys:

4 $$ {\text{AUC}} = \sum\limits_{\text{i}}^{\text{decoys}} {\Updelta {\text{FPR}}\;{\text{TPR}}_{\text{i}} } = \frac{1}{{{\text{N}}_{\text{decoys}} }}\sum\limits_{\text{i}}^{\text{decoys}} {{\text{TPR}}_{\text{i}} } = \left\langle {\text{TPR}} \right\rangle_{\text{decoys}} = 1 - {\text{FPR}}_{\text{actives}} $$

where TPR_i_ is the true positive rate at decoy *i* and ΔFPR is the constant increment in the false positive rate. The difference between the AUC values of the pair of receptor models A and B becomes as Eq. 5:

5 $$ \Updelta {\text{AUC}} = {\text{AUC}}_{\text{A}} - {\text{AUC}}_{\text{B}} = \left\langle {\text{TPR}} \right\rangle_{\text{decoys,A}} - \left\langle {\text{TPR}} \right\rangle_{\text{decoys,B}} = \left\langle {{\text{TPR}}_{\text{A}} - {\text{TPR}}_{\text{B}} } \right\rangle_{\text{decoys}} $$

where the last step arose from docking of the same library into all receptor models, which statistically indicates the pairing of samples. As a result,

6 $$ \begin{aligned} \Updelta {\text{AUC}} & = \frac{1}{{{\text{N}}_{\text{decoys}} }}\sum\limits_{\text{i}}^{\text{decoys}} {\left( {{\text{TPR}}_{{{\text{i}},{\text{A}}}} - {\text{TPR}}_{{{\text{i}},{\text{B}}}} } \right) = \left\langle {{\text{TPR}}_{\text{A}} - {\text{TPR}}_{\text{B}} } \right\rangle_{\text{decoys}} } \\ & = \frac{1}{{{\text{N}}_{\text{actives}} }}\sum\limits_{\text{i}}^{\text{actives}} {\left( {{\text{FPR}}_{{{\text{i}},{\text{B}}}} - {\text{FPR}}_{{{\text{i}},{\text{A}}}} } \right) = \left\langle {{\text{FPR}}_{\text{B}} - {\text{FPR}}_{\text{A}} } \right\rangle_{\text{actives}} } \\ \end{aligned} $$

Then the variances for the actives and decoys are given by:

7 $$ {\text{Var}}_{{\Updelta ,{\text{a}}}} = \frac{1}{{{\text{N}}_{\text{actives}} }}\sum\limits_{\text{i}}^{\text{actives}} {\left\{ {({\text{FPR}}_{{{\text{i}},{\text{A}}}} - {\text{FPR}}_{{{\text{i}},{\text{B}}}} ) - \left\langle {{\text{FPR}}_{\text{A}} - {\text{FPR}}_{\text{B}} } \right\rangle_{\text{actives}} } \right\}^{2} } $$

8 $$ {\text{Var}}_{{\Updelta ,{\text{d}}}} = \frac{1}{{{\text{N}}_{\text{decoys}} }}\sum\limits_{\text{i}}^{\text{decoys}} {\left\{ {({\text{TPR}}_{{{\text{i}},{\text{A}}}} - {\text{TPR}}_{{{\text{i}},{\text{B}}}} ) - \left\langle {{\text{TPR}}_{\text{A}} - {\text{TPR}}_{\text{B}} } \right\rangle_{\text{decoys}} } \right\}^{2} } $$

with the standard error in ΔAUC given as:

9 $$ {\text{SE}}_{\Updelta } = \sqrt {\frac{{{\text{Var}}_{{\Updelta ,{\text{a}}}} }}{{{\text{N}}_{\text{actives}}  }} + \frac{{{\text{Var}}_{{\Updelta ,{\text{d}}}} }}{{{\text{N}}_{\text{decoys}} }}} $$

Finally, the two-sided *p* value for ΔAUC between the two receptors is obtained as a Gaussian distribution with a standard deviation equal to SE_Δ_:

10 $$ {\text{p}} = {\text{erfc}}\left( {\frac{{\left| {\Updelta {\text{AUC}}} \right|}}{{\sqrt 2 {\text{SE}}_{\Updelta } }}} \right) $$

where erfc is the complementary error function. All analyses of the receptor predictive power were done with Matlab R2011a (Version 7.12.0.635) [43].

### pKa prediction analysis

PROPKA [5–8], Maestro [28, 44], H++ [9–11], and MCCE [12–14] were employed to predict the protonation states of His62 and His119 in the active site of RmlC. Along with the dimer of chains A and B from the homodimeric holo structure (PDB ID: 2IXC [27]), we also generated an additional structure by removing the bound TRH molecule to examine the effect of the ligand on pKa prediction. Therefore, the dimers with and without the ligand were subjected to the pKa calculation with PROPKA, Maestro, H++, and MCCE.

PROPKA predicts the pKa by solving the linearized Poisson–Boltzmann equation [5–8]. The algorithm calculates a pKa shift, ΔpKa, arising from perturbation of electrostatic energy of an ionizable residue between its charged neutral states. Thus the pKa is predicted by:

$$ {\text{pKa}} = {\text{pK}}_{\text{Model}} + \Updelta {\text{pKa}} $$

with additional terms and parameters describing the Coulomb interaction, desolvation, unfavorable electrostatic reorganization energies, and hydrogen bonding networks. The model pKa used for histidine in PROPKA is 6.50.

The Protein Preparation Wizard of Maestro [28, 44] has been updated to employ PROPKA by default, from its previous version using Epik [31, 32]. The pKa calculation with Epik relies on the well-established Hammett and Taft (HT) [45] linear free energy approach and the quality of hydrogen bonding networks. In this study, we compared the pKa prediction of Maestro both with and without PROPKA.

H++ is a single-structure continuum electrostatics methodology that predicts the pKa values of the titratable residues based on either Generalized Born or Poisson–Boltzmann method using the AMBER 10 force field [9–11]. Multi-conformation continuum electrostatics (MCCE) calculates the pKa values of the ionizable protein residues and ligands by generating rotamers throughout a titration with Monte Carlo sampling [12–14]. The changes in the conformation create a position dependent dielectric response and the degrees of freedom of the conformers are added to calculate the Boltzmann distribution of the ionization states and atomic positions. The pairwise electrostatic interactions between different conformers are calculated by the DelPhi Poisson–Boltzman solver.

## Results and discussion

In order to evaluate the effect of histidine protonation and rotameric states on the predictive performance of receptors, we performed virtual screening (VS) for the *Mtb* enzyme RmlC based on the results of a previous high-throughput screening (HTS) study. Below, we will first examine the typical interactions of the co-crystal ligand TRH to probe the ligand pose dependence on histidine protonation. We further contextualize analysis of enrichment performance and predictive power of various receptor models, by discussing the interactions with the receptor to show the effect of different histidine protonation states on VS. Finally, we compare the predicted pKa values calculated by several common pKa calculation packages to the receptor protonation states with the best predictive power.

### Docking of TRH

Docking the co-crystal ligand TRH back into 36 receptor models was carried out to show the pose, or ligand orientation relative to the receptor, dependence on histidine protonation and rotameric states. Chemically intuitive hydrogen bonding patterns for the crystal coordinates of His62 and His119, shown in Fig. 2b, imply the potential significance of hydrogen bonds in docking of TRH. Docking this ligand allowed for preliminarily examination of the dependence of pose on the possible hydrogen bonding networks with the receptor.

Varying histidine protonation states has a clear effect on pose prediction for the determined co-crystal ligand. RMSD of docking pose of self-docked TRH into the crystal coordinates for different protonation and rotameric states of His62 and His119 varied from 2.91 to 5.44 Å. The protonation state of both histidines with the best average RMSD is HIE, which agrees with the most probable protonation states of the crystal coordinates of TRH. Also, in all cases the docking algorithm predicts the position of the pyrophosphate of the ligand correctly, but the large deviation from the crystal coordinates mainly stems from the flipping of the thymidine and rhamnose moieties around the pyrophosphate, resulting in different hydrogen bonding patterns between TRH and two histidines. This indicates the importance of hydrogen bonding networks with His62 and His119 in the pose prediction of the co-crystal ligand TRH. Therefore, after examining the pose dependence upon hydrogen bonds provided by two histidines, we expanded our study to look systematically at ranking compounds in VS and how it is affected by the protonation and rotameric states of histidines.

### Virtual screening

Molecular docking was carried out to examine compound ranking dependence on histidine protonation and rotomeric states. The ligand set included ten actives and 2,000 inactives selected at random from a HTS. We note that Tanimoto scores indicate that most of our decoys have a low similarity to the actives. Such a decoy set presents a smaller challenge to the docking algorithm and the predictive performance of VS itself may be affected when decoys with greater similarity to the actives are used. However, this study aimed to examine not the predictive performance of the docking algorithm per se, but how histidine protonation states affect the relative performance in VS.

**Fig. 3:**
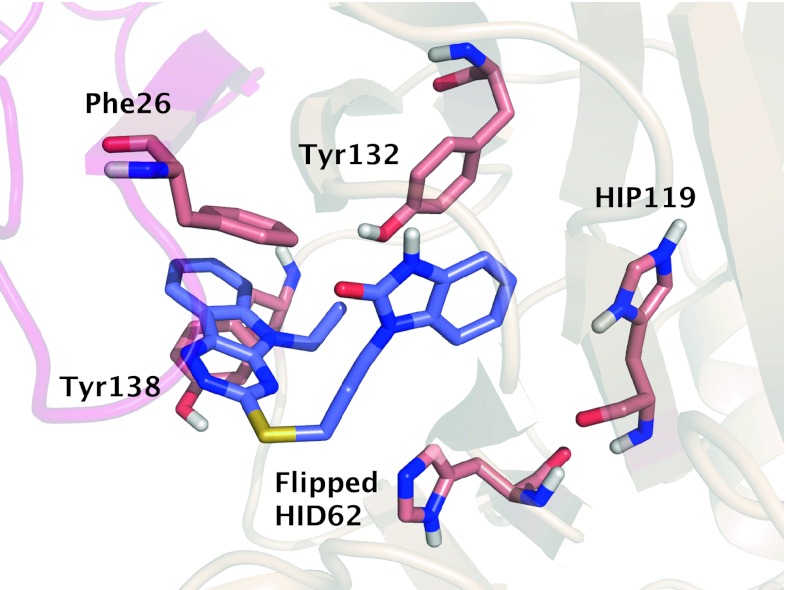
Predicted interaction of the initial hit compound SID7975595 with flipped HID62 and HIP119 in receptor model 23. Generally, the actives do not have strong interactions with His62 or His119, yet varying histidine protonation states have a profound effect on the ranked results. Favorable interactions are observed with other binding site residues, such as Tyr132 and Tyr138 as depicted here

Docked active ligands and the product analog were examined initially to characterize important interactions in the RmlC binding site. In all receptor models, hydrophobic pi–pi stacking interactions contribute significantly to docking score of the active compounds within the RmlC active site. The initial hit compound from HTS, SID7975595, is ranked high in most receptor models, between 8th and 51st rank in 26 out of 36 receptors. Although there is only limited structural similarity between SID7975595 and the co-crystal ligand TRH, the tricyclic ring of SID7975595 readily replaces the TRH thymidine moiety, while the benzimidazolone ring replaces the rhamnose moiety, providing structural basis of the inhibition. As shown in Fig. 3, the hydrophobic interaction between the actives and receptor often involves Tyr132 and Tyr138 from chain A and Phe26 from chain B (note that a part of chain B intrudes in the active site of chain A). Through interacting with the essential binding site residues and preventing water molecules from accessing Phe26 and Tyr132, the actives provide abundant hydrophobic contacts to achieve the high binding affinity. As discussed in Sivendran et al. [16], substitution of the ethyl group attached to the nitrogen on the tricyclic ring of SID7975595 by an allyl group (e.g., the active compound 77074) further enhances the binding affinity by forming an even tighter hydrophobic seal. In comparison, substitution of this group by a smaller methyl group or a hydrogen atom results in a lower binding affinity [16]. In addition to the hydrophobic contacts described above, some of the actives also form hydrogen bonds with Ser51, Arg59, and Arg170. A figure describing the interactions of the docked actives can be found in Online Resource 3.

Interestingly, the actives generally do not achieve polar interactions with His62 and His119. As shown in Fig. 3, the carbonyl oxygen and two benzimidazolone nitrogens of SID7975595 face away from His62 and His119. The direction of aromatic hydrogens of the actives is often unable to participate in hydrogen bonding networks with the two histidines. Nevertheless, different protonation and rotameric states of these histidines do affect the VS results through their interactions with the decoys.

### Assessment of differences in ranking

It is not uncommon that only the top 1 % of compounds screened can be tested experimentally in a VS study, due to the limited resources. Therefore, the enrichment factor (EF)^1%^ metric, which reflects the database enrichment performance in the top 1 % (20 docked compounds) of a library, becomes particularly relevant in assessing the predictive power of VS. The EF^1%^ ranges from 0 to 80 for 36 receptor models (Table 1), indicating that the VS results are sensitive to the protonation and rotameric states of His62 and His119 of RmlC. Nevertheless, 28 out of 36 receptors rank more than eight actives within the top 10 % in the VS, as reflected by the EF^10%^ (Table 1), suggesting that most receptors are able to distinguish the actives and decoys when a larger portion (10 %) of the database is considered. The EF results also suggest that the receptor models with HIP62 or HIP119 tend to have poor enrichment performance, likely due to the extensive hydrogen bonding networks with the decoys, as discussed later.

**Fig. 4:**
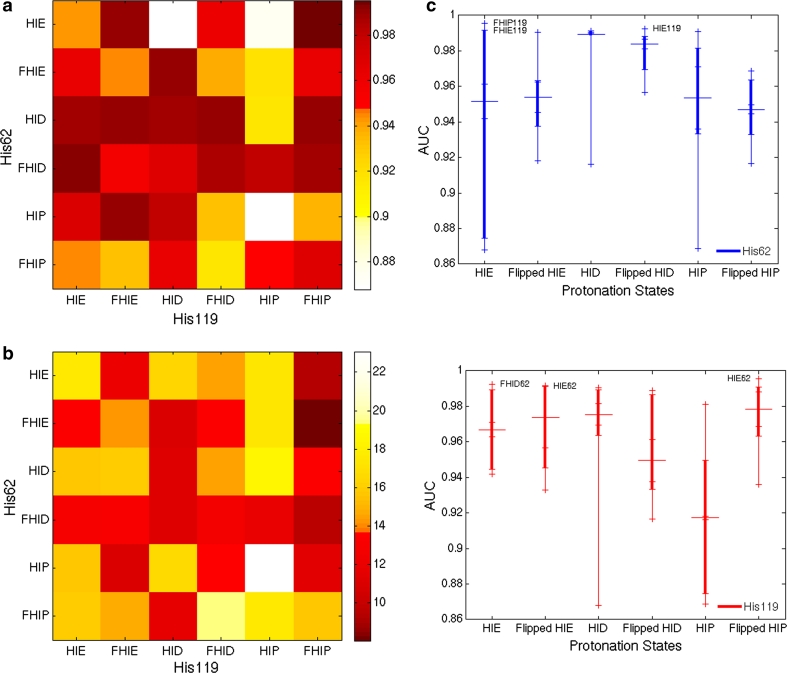
**a** AUC values of 36 receptor models. Protonation and rotameric states are marked for each histidine. Flipped states are marked with the *letter F*. *Darker color* indicates higher AUC and better predictive performance of the corresponding receptor model. **b** Average hydrogen bond percentage of the top 1 % compounds in 36 VS runs. Protonation and rotameric states are marked for each histidine. *Lighter color* indicates higher hydrogen bond percentage, with % unit for the *colorbar*. The R^2^ for the correlation between the AUCs and average hydrogen bond percentage for each VS run is 0.42 (see Online Resource 4 for the scatter plot). **c** Receptor performance dependence on His62 (*top*) and His119 (*bottom*). The median of the AUC values of each protonation state is shown with *large horizontal line*. The *small ticks* in each histidine model mark six different protonation states of the other histidine. The *thicker vertical lines* represent 25–75 % range of the AUCs. The best receptor models are shown explicitly with the models’ protonation states

The area under the receiver operating characteristic curve (AUC) for each receptor model was evaluated to report the enrichment performance of models upon different protonation and rotameric states of His62 and His119. As shown in Fig. 4a and Table 1, the AUC values of all receptor models range from 0.868 to 0.996, indicating an overall good predictive performance (an AUC of 0.5 corresponds to no differentiation between the actives and decoys). In general, the AUC result is complementary to the EF assessment for receptor predictive performance. Summarizing Table 1, Fig. 4c shows how the range of the receptor performance depends on the two histidine protonation and rotameric states. Considering the 25–75 % range of the AUCs (Fig. 4c, indicated by the thicker lines), the His62 models show a larger variation across His119 states. The His119 models, on the other hand, have a more consistent performance regardless of the protonation states of His62, with the exception of HIP state. This indicates that different protonation states of His62 have a smaller influence than those of His119 on the receptor performance in our screening.

A stronger dependence of enrichment on the protonation states of His119 is observed in the HIE62 and HIP62 models. With HIE62 state, the models with flipped HIP119 (model 6) and flipped HIE119 (model 2) yield the highest receptor performance. Models 3 and 5 with HID119 and HIP119, respectively, lead to the worst enrichment. In examining why HIE62 state has the largest variation in AUCs, one finds that His62 has either pi–pi stacking or no interactions with ligands, and makes only a few hydrogen bonds with high-ranking decoys. Therefore, the receptor performance depends on the interaction of His119 with the decoys. This is also seen when examining the broad performance range of the AUCs of the HIP62 models. The hydrogen bonding networks with the decoys will be discussed later in the following section.

In order to evaluate the statistical significance of difference of the AUC values between a pair of receptor models, we performed a two-sided *p* test at the 95 % level on the null hypothesis that the pair has statistically comparable AUC values, against the alternative hypothesis that their difference in the AUC values and predictive power is statistically meaningful. The result is shown in Online Resource Table 1, with the *p* values less than 0.05 emphasized. On average, the receptors have more than 16 *p* values less than 0.05, demonstrating the sensitivity of VS on histidine protonation and rotameric states. As one might expect, the receptors with the most significant differences correspond to the models with the highest (model 6) or lowest AUC values (models 3, 29, and 5). Model 6 is statistically better at ranking the actives over decoys than 26 other receptors in the ensemble. Models 3, 29, and 5 are distinguishably worse at ranking the actives than 29, 25, and 31 other receptors, respectively.

Quantitative analysis of the hydrogen bonding interactions was carried out for the top 1 % (20 docked compounds) of each VS result to account for the abundant hydrogen bonding interactions with the binding site residues often observed with the decoys. The results indicate an inverse correlation between the hydrogen bonding contribution and receptor performance. Figure 4b shows the average hydrogen bond percentage of each receptor model for the top 1 % docked compounds. Hydrogen bond percentage is defined as the portion of the Glide XP hydrogen bonding term in the total docking score. Comparison of Fig. 4a, b reveals the inverse relationship between the hydrogen bond percentage and AUC with a R^2^ of 0.42 (y = −56.18x + 67.95, the correlation is plotted in Online Resource 4). The inverse relationship is commonly observed with models with HIP119, flipped HIP119, or HID62, where the high hydrogen bond percentage resulted in poor enrichment. For example, receptor model 29 with HIP62 and HIP119, where both histidines presenting active site facing hydrogen bond donors, has one of the worst AUCs due to the high percentage of hydrogen bonds in the top hits.

Notably, the hydrogen bonding potential of His119 often determines the receptor performance. For example, the model with HID62 and HIP119 was an outlier among the HID62 models in Fig. 4c, with noticeably low enrichment compared to the overall good performance of the other five HID62 models. The HID62 models have a high median AUC of 0.989, despite the frequent hydrogen bonding to the decoys from HID62. This is due to His119 states achieving few hydrogen bonding interactions with the decoys. Only with HIP119 state does the HID62 model make hydrogen bonds with a number of decoys, resulting in the relatively low AUC. This observation agrees with the stronger dependence of the receptor performance on the protonation states of His119, as discussed above. Online Resource 4 describes the AUC distribution and hydrogen bond percentage along with the direction of hydrogen bond donor or acceptor from two histidines facing the receptor.

Above analyses highlight the hindering effect of hydrogen bonding to the decoys on the predictive power of VS, due to the various coordinates of two histidines with different protonation and rotameric states. The scatter of the observed correlation with the R^2^ of 0.42 is likely attributed to several causes, including the chemical nature of the decoy dataset, as well as the slight differences in geometry of each receptor upon minimization in the initial preparation of the protein. By clearly showing the sensitivity of virtual screening results on different protonation and rotameric states of histidines in the active site, we emphasize that care should be taken when preparing the atomic coordinates of a receptor for VS, particularly considering the general properties of the ligands being screened. This includes taking into account the hydrogen bonding to the co-crystal ligand and its effect on protein preparation, as well as a comprehensive analysis of proximal hydrogen bonding networks. This is usually achieved by examining the results from widely used pKa prediction software packages, and to this point, we have compared results from different packages relative to our VS results and discuss them further.

### Docking of the decoys

Various factors lead to differences in ranking across the receptors, particularly with respect to the decoys. Generally, the decoys that ranked higher than the actives were of high molecular weight and had more potential to have hydrogen bonds with the receptor. In this section, we further analyze the frequent interaction patterns observed between the decoys and receptor, with a focus on the receptor models with poor enrichment.

**Fig. 5:**
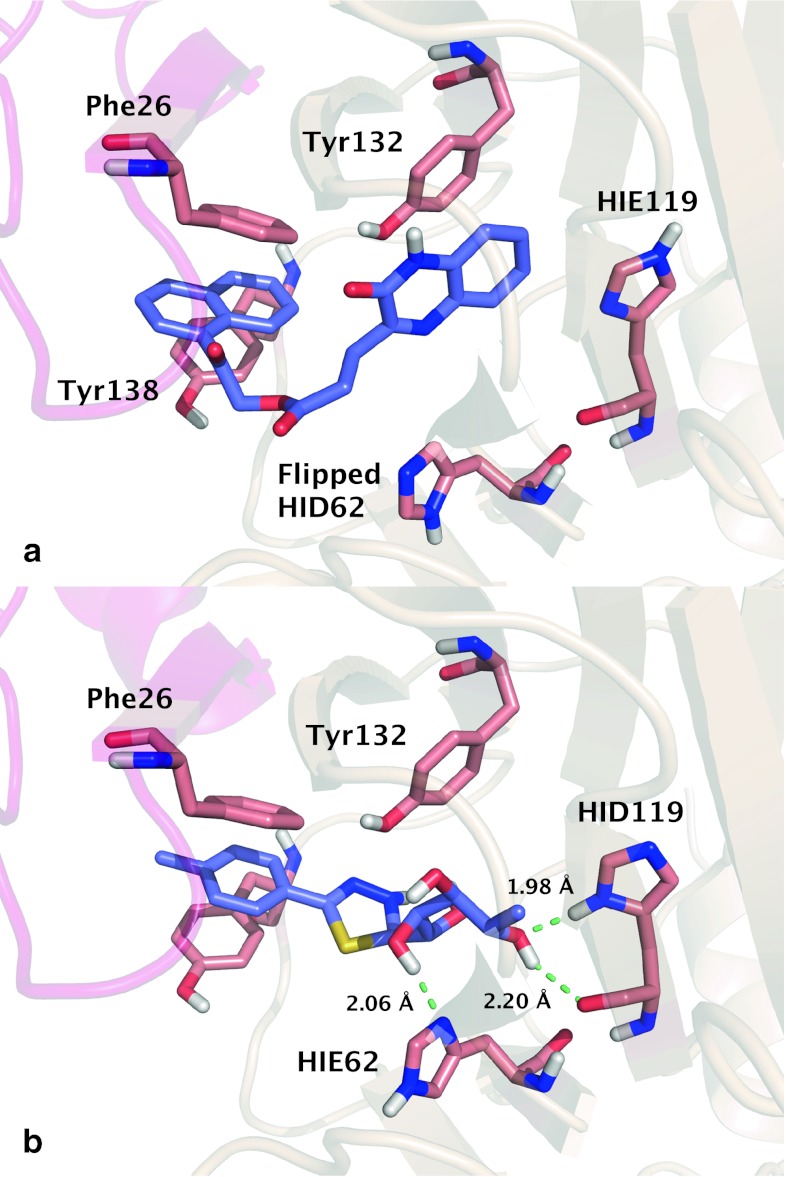
**a** Interaction of the inactive compound 16952387 with flipped HID62 and HIE119 in receptor model 19. The compound has no interaction with either histidine. Pi–pi stacking interactions with Phe26 from chain B, Tyr132, and Tyr138 contribute to its high rank, along with hydrogen bonds with Arg23, Arg59, Arg170, and Ser51 (not shown). **b** Interaction of the inactive compound 17388064 with HIE62 and HID119 in receptor model 3. Both histidines provide hydrogen bonds to the compound

Decoys tend to have larger molecular weight and more ring structures than the actives (Table 2). This results in the decoys ranking higher, due to hydrophobic interactions in the absence of hydrogen bonds to the receptor. Figure 5a shows the hydrophobic interactions achieved through the large inactive compound 16952387 in receptor model 19. This compound is often ranked within the top five in many VS runs for its substantial pi–pi stacking interactions with Phe26, Tyr132, and Tyr138. This trend is frequently observed in virtual screening where larger molecules rank better as a result of extensive interactions with the receptor [46].

**Table 2:** Comparison of molecular weight, number of hydrogen bond donor, and number of hydrogen bond acceptor for the actives and decoys

	Actives	Decoys
Average molecular weight (g/mol)	417.69	353.85
Stdev. of molecular weight (g/mol)	27.07	80.85
Average number of hydrogen bond donor	1.1	1.04
Average number of hydrogen bond acceptor	5.8	5.89

The enrichment performance is particularly low for the receptors providing abundant hydrogen bonding networks to the decoys. Interactions through His62 and His119 were not widely observed for the actives, and therefore compounds with larger enthalpic contributions erroneously rank more favorably. An example shown in Fig. 5b depicts the interaction of inactive compound 17388064 in receptor model 3 (AUC 0.868), ranked as first. In this receptor, which is the worst at ranking compounds based on AUC, compound 17388064 forms two hydrogen bonds with two histidines, one between its hydroxyl hydrogen and the δ-nitrogen of HIE62 and the other between its hydroxyl oxygen and the hydrogen on δ-nitrogen of HID119. This compound has five hydrogen bond donors and nine acceptors, a large number compared to the respective averages of those of the decoys and actives (Table 2). Therefore, with a high hydrogen bonding contribution to the total score of 34.7 ± 6.62 %, this decoy compound is frequently observed to form at least one hydrogen bond with either of the two histidines, thereby achieving high ranks in multiple VS runs.

Two other receptor models, model 29 with HIP62 and HIP119 and model 5 with HIE62 and HIP119, show similar interaction patterns to decoys as model 3. These three models have the lowest AUC values, with an average of 0.870 among them. As discussed above, their AUC values differ significantly from other receptors, reflecting the subtle relationship between hydrogen bonds achieved through His62 and His119 and poor enrichment. An additional figure describing the hydrogen bonding networks between the decoys and receptors is provided in Online Resource 5.

### pKa prediction for His62 and His119

Our results clearly demonstrate the sensitivity of virtual screening on histidine protonation and rotameric states. In many computational biophysical studies, the protonation states of the titratable residues are determined using various pKa prediction programs. To assess the performance of these programs to identify the receptor model with the best predictive power in docking, we compared the pKa prediction results of His62 and His119 from PROPKA, Maestro, H++, and MCCE, as shown in Table 3 with the calculated pKa values.

**Table 3:** Comparison of the predictions for protonation states of His62 and His119 of RmlC made by commonly used software, with calculated pKa values

	RmlC-TRH complex		RmlC without TRH	
	His62	His119	His62	His119
PROPKA 3.1				
pKa	4.24	5.8	5.16	6.12
Protonation	Neutral	Neutral	Neutral	Neutral
Maestro with PROPKA				
pKa	7.09	5.6	4.99	6.12
Protonation	HIP	HIE	HIE	HIE
Maestro with Epik				
Protonation	HIP	HIP	HIP	HIP
H++				
pKa	3	<0.0	3	<0.0
Protonation	HIE	HIE	HIE	HIE
MCCE				
pKa	<0.0	7.201	2.771	1.347
Protonation	Neutral	HIP	Neutral	HIP

First, PROPKA 3.1 predicts that both His62 and His119 are neutral regardless of the presence of TRH during preparation. The program, however, cannot assign rotameric states of histidines. Therefore, a state of HID, flipped HID, HIE, or flipped HIE must be determined manually. Similar to PROPKA, the program H++, which uses a single-structure continuum electrostatics, also finds both histidines to be neutral, although the predicted pKa values are different from those from PROPKA. The program MCCE, which is based on multi-conformation continuum electrostatics, predicts His62 to be neutral while His119 to be protonated.

Next, we used the Protein Preparation Wizard in Maestro to calculate pKa of His62 and His119 with and without TRH. Note that Maestro is able to vary rotameric states, whereas PROPKA cannot. A recent update enables Maestro to employ PROPKA in its pKa prediction instead of Epik. With Epik, Maestro predicts both His62 and His119 in doubly protonated states, regardless of the presence of TRH. Interestingly, the receptor model that corresponds to this multi-histidine state has the worst predictive power with an AUC of 0.869. When PROPKA is used, HIP62 and HIE119 are predicted for the protein-TRH complex and HIE62 and HIE119 for the apo protein. These two predictions by PROPKA in Maestro correspond to the models of moderate enrichment performance, with AUCs of 0.971 for model 25 (HIP62 and HIE119) and 0.942 for model 1 (HIE62 and HIE119), respectively.

Given that the above predictions made by different software vary significantly from each other, caution should be taken when using these results as a guideline to prepare a protein for virtual screening. Without intimate knowledge of the true protonation state of the receptor as well as the ligands being screened, it is difficult to address this problem. Therefore, we suggest that a small-scale analysis, like one performed in this study, and comparison with experimental data, if available, could provide a more accurate description of protonation and rotameric states of the titratable residues in protein receptors for future larger-scale screenings. Alternatively, a model that includes explicit incorporation of alternative side chain protonation and rotameric states during docking, potentially with information stored in the grid as exists for rotatable hydroxyls and thiols in Glide, may be worth pursuing. Examination of the results with respect to the protonation states and rankings based on interactions with histidines should be carefully examined before proceeding to experimental testing.

Additionally, receptor flexibility will likely affect the protonation states of the ionizable residues. While this was not explicitly studied here, aside from minimization of each receptor after assigning protonation states, protein flexibility is clearly important for drug design and development [47, 48]. Considering conformational and protonation space in conjunction becomes quickly intractable with physical methods such as those described here, but enhanced sampling methods show promise in tackling such difficulties [49]. This includes constant pH molecular dynamics simulations, for which the pH is an external thermodynamic variable, used for blind prediction of pKa values of the titratable residues [50–52]. Effectively applying results from these simulations to molecular design is an ongoing area of interest. Equilibrium ensembles from such simulations can be used in conjunction with docking as an application of relaxed complex scheme, where virtual screening is conducted with an ensemble of differently protonated structures, to improve the enrichment results [53]. Taking receptor flexibility into account in the target preparation will lead to broader sampling of conformational and protonation space, thus enhancing the performance of VS.

## Conclusions

Protein–ligand recognition is of central importance in structure-based drug discovery. Correctly accounting for the chemical environment surrounding the ligand is imperative for characterizing and predicting the molecular interactions. The possible effect of the various protonation and rotameric states of the ionizable residues of the receptor on virtual screening (VS) is critical yet often overlooked. In this study, we thoroughly examined the influence of the protonation and rotameric states of histidine on the predictive power of the docking protocol for drug discovery.

A histidine can adopt three different forms depending on the net charge and the location of proton(s). Due to common ambiguities in X-ray crystal structures, three additional states may be generated through flipping of the imidazole ring. In this work, we performed a VS study on the *Mtb* enzyme RmlC to investigate the effect of six protonation and rotameric states of histidines. We systematically examined the contribution of hydrogen bonding interactions provided by two histidines in the active site, His62 and His119. The predictive performance of receptors was assessed by quantitatively analyzing enrichment factors and area under the receiver operating characteristic curve. We showed that the hydrogen bonding networks involving His62 and His119 are important in the interaction between the co-crystal ligand TRH and active site, validating the significance of accurate description of protonation and rotameric states of the two histidines in VS. We compared the typical patterns of interactions achieved with the active site residues observed for the active compounds and decoys; whereas the actives often involve only hydrophobic interactions, the high-ranking decoys are erroneously enriched by additional hydrogen bonds provided by His62 and His119. Our analyses reveal the sensitivity of virtual screening on protonation, ionization, and rotameric states of active site histidines. We recommend a priori analysis of receptor-ligand hydrogen bonding interactions, in addition to the usage of protonation assignment software packages, to prepare a receptor for virtual screening. Systematically assessing binding site protonation state effects before conducting a large virtual high-throughput screening, beyond empirical state prediction, may therefore result in enrichment gains.

## Electronic supplementary material

Below is the link to the electronic supplementary material.

Supplementary material 1 (PDF 14918 kb)

Supplementary material 2 (PDF 109 kb)
