# Supplementary material for: Effects of histidine protonation and rotameric states on virtual screening of M. tuberculosis RmlC
Source: J Comput Aided Mol Des. 2013 Apr 12;27(3):235–46. doi: 10.1007/s10822-013-9643-9 (PMC3639364; doi:10.1007/s10822-013-9643-9)
Supplement: Supplementary file 1 — Supplementary material 1 (PDF 14918 kb) [file 10822_2013_9643_MOESM1_ESM.pdf]

Supplementary Material (Figures)

Article title:
Effects of Histidine Protonation and Rotameric States on Virtual Screening of *M. tuberculosis* RmlC

Journal name:
Journal of Computer-Aided Molecular Design

Authors:
Meekyum Olivia Kim[1,*], Sara E. Nichols[1,2,3], Yi Wang[4,*,†], J. Andrew McCammon[1,2,3,4]

[1] Department of Chemistry and Biochemistry, University of California San Diego, La Jolla, CA 92093, USA
[2] Department of Pharmacology, University of California San Diego, La Jolla, CA 92093, USA
[3] Center for Theoretical Biological Physics, University of California San Diego, La Jolla, CA 92093, USA
[4] Howard Hughes Medical Institute, University of California San Diego, La Jolla, CA 92093, USA

* To whom correspondence should be addressed:
Meekyum Olivia Kim, mok007@ucsd.edu;
Yi Wang, yiwang@phy.cuhk.edu.hk

† Current address: Department of Physics, The Chinese University of Hong Kong, Shatin, N.T., Hong Kong

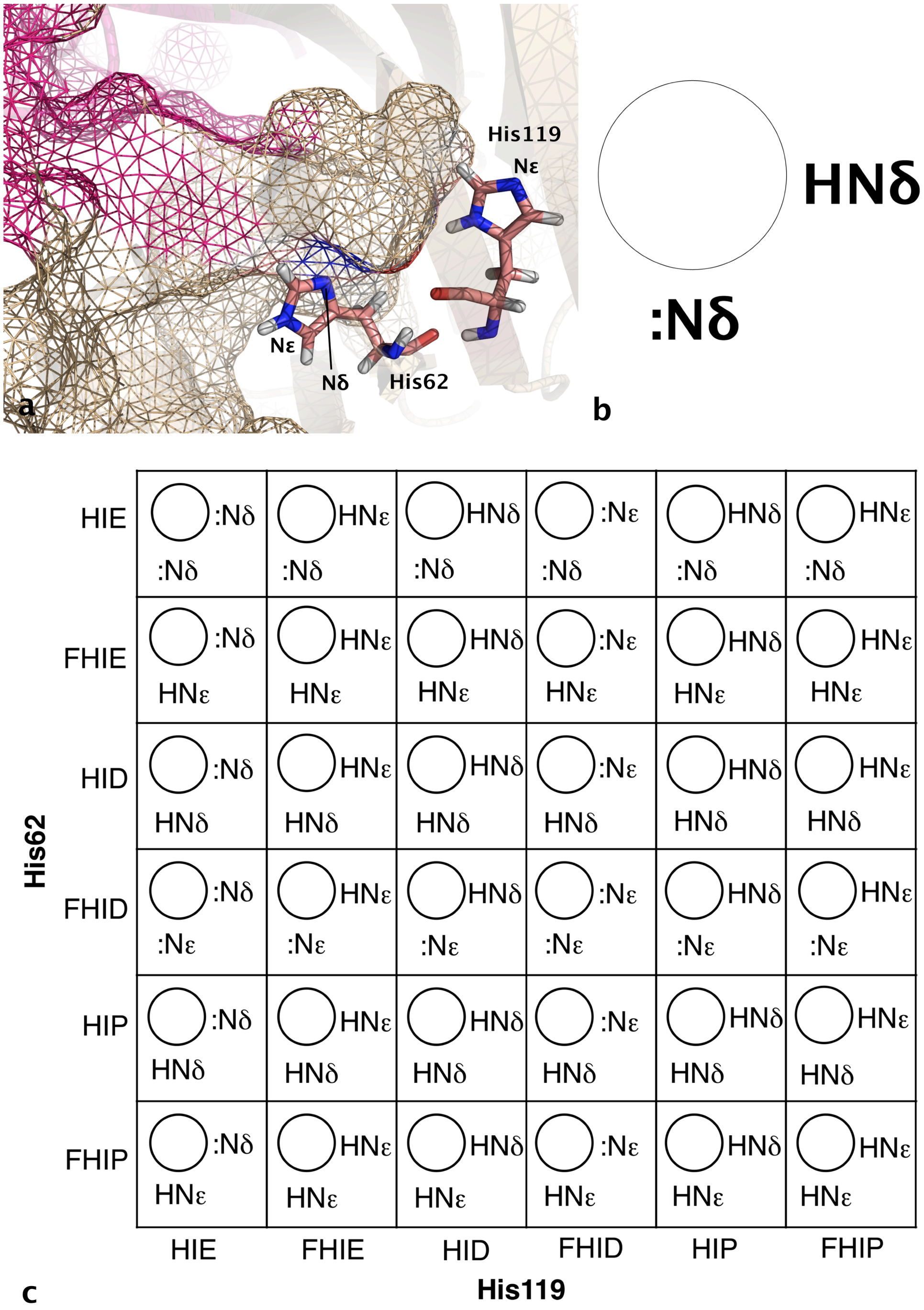

Supplementary Fig. 1 (a) Location of His62 and His119 with respect to the active site of RmlC. Atom types of nitrogen of histidine are shown as either Nδ or Nε and the surface is represented as wireframes.
(b) Schematic description of hydrogen and nitrogen of two histidines in (a). The active site is shown as a circle and His62 is located below the circle and His119 right next to it. Colon (:) represents a lone pair of electrons on nitrogen.
(c) Schematic description of hydrogen and nitrogen of two histidines in 36 receptor models following (b)

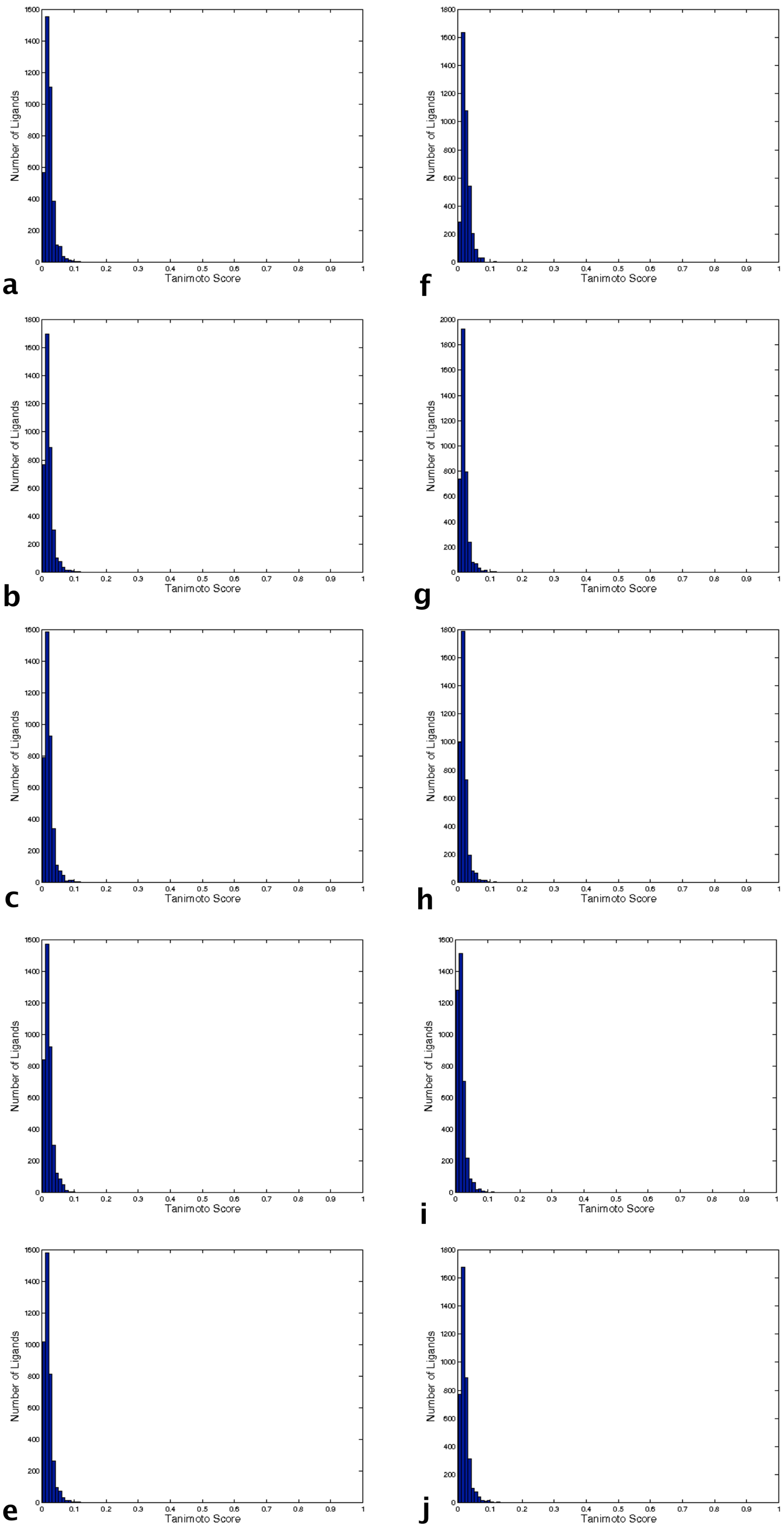

a
b
c
d
e
f
g
h
i
j
Number of Ligands
Tanimoto Score

Supplementary Fig. 2 Distribution of Tanimoto scores of the library of total 3,934 compounds against each of ten active compounds as a reference. The active compound used to calculate Tanimoto score is (a) 77070, (b) 77071, (c) 77072, (d) 77073, (e) 77074, (f) 78531, (g) 78532, (h) 78533, (i) SID7972845, and (j) SID7975595, respectively.

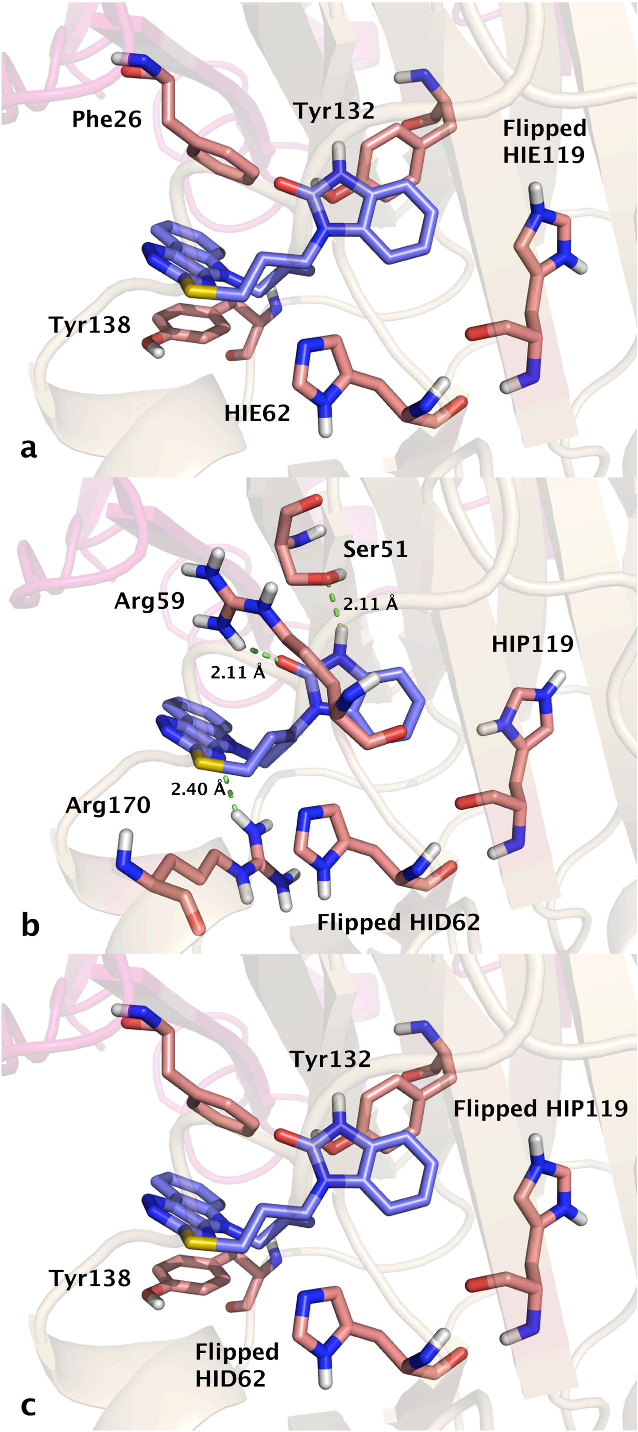

Supplementary Fig. 3 (a) Predicted interaction of the active compound SID7975595 with HIE62 and flipped HIE119 in receptor model 2 (AUC 0.992). The compound mainly has hydrophobic interactions.
(b) Predicted hydrogen bonding networks between the active compound SID7975595 and Arg59, Arg170, and Ser51 in the active site in receptor model 23 with flipped HID62 and HIP119 (AUC 0.981)
(c) Interaction of the active compound 77074 with flipped HID62 and flipped HIP119 in receptor model 24 (AUC 0.988). The compound 77074, where the ethyl group attached to the nitrogen on the tricyclic ring of the compound SID7975595 has been substituted by an allyl group, has the lower IC_{50}. Hydrophobic pi-pi stacking comprises the main interaction between the compound and the active site residues.

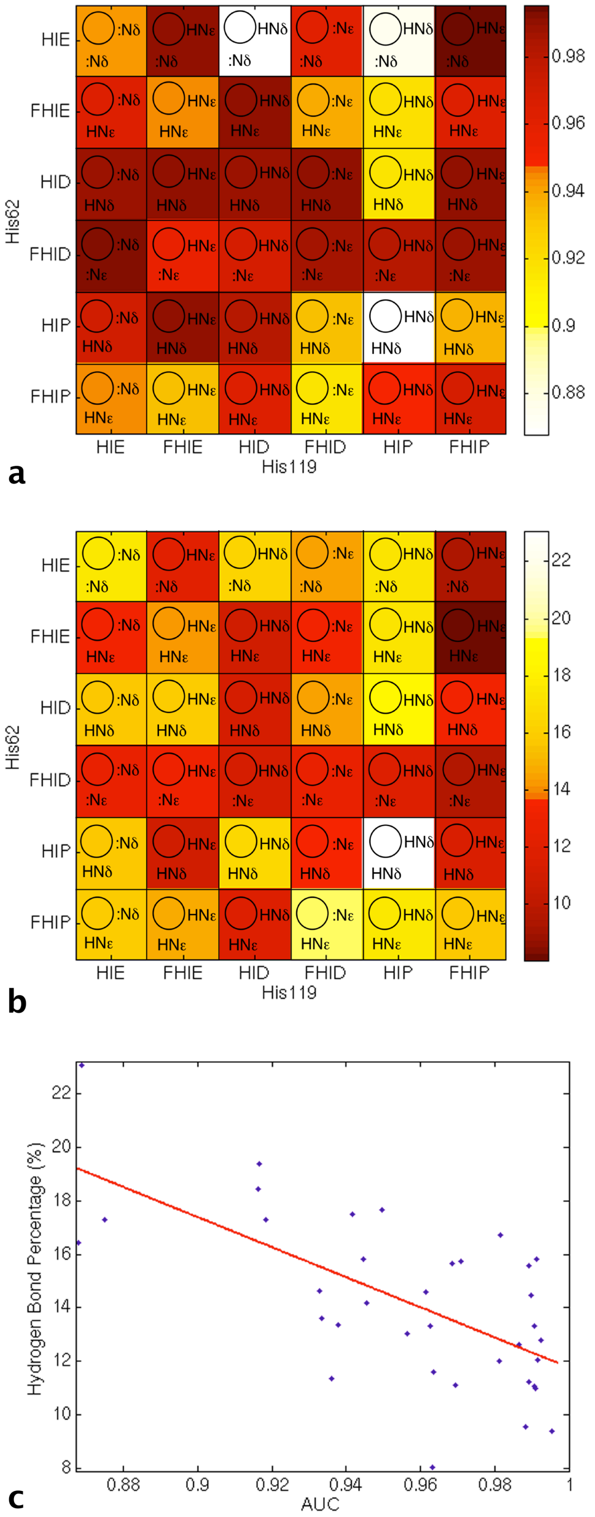

Supplementary Fig. 4 (a) AUC values of 36 receptor models (Fig. 4a) shown with hydrogen bond donor or acceptor of two histidines for each model as described in Supplementary Fig. 1

(b) Average hydrogen bond percentage of the top 1% compounds in 36 VS runs (Fig. 4b). Hydrogen bond donor or acceptor from two hisidines is shown together in the same way as in (a).

(c) Scatter plot showing the correlation between the AUCs and average hydrogen bond percentage for the top 1% compounds of each VS run. The correlation is observed as y = - 56.18x + 67.95 with the R^2 of 0.42.

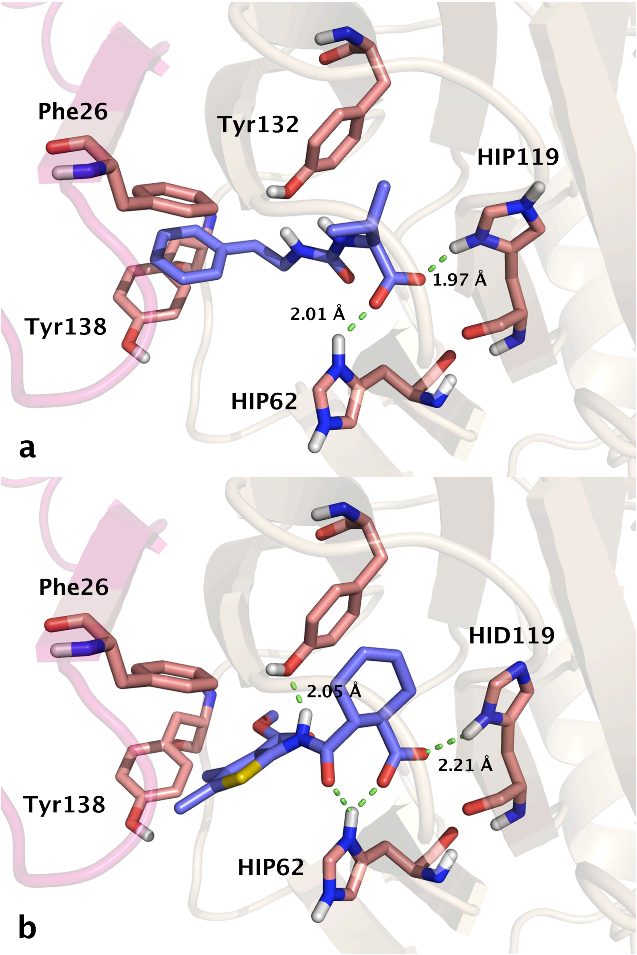

Supplementary Fig. 5 (a) Interaction of the inactive compound 14741063 with HIP62 and HIP119 in receptor model 29 (docking score -8.513, AUC 0.869). Both hydrogens on δ-nitrogens of HIP62 and HIP119 accept lone electron pairs from carboxylic acid group of the compound. Phe26 and Tyr132 interact with the compound via pi-pi stacking interaction.

(b) Interaction of the inactive compound 14736762 with HIP62 and HID119 in receptor model 27 (AUC 0.982). Hydrogen on δ-nitrogen of HIP62 makes two hydrogen bonds with the amide oxygen and carboxylic oxygen of the compound while that of HIP119 also forms a hydrogen bond with carboxylic oxygen. Additional hydrogen bonding networks include the one between the hydroxyl oxygen of Tyr132 and the amide nitrogen of the compound.
