# Supplementary material for: Effects of histidine protonation and rotameric states on virtual screening of M. tuberculosis RmlC
Source: J Comput Aided Mol Des. 2013 Apr 12;27(3):235–46. doi: 10.1007/s10822-013-9643-9 (PMC3639364; doi:10.1007/s10822-013-9643-9)
Supplement: Supplementary file 2 — Supplementary material 2 (PDF 109 kb) [file 10822_2013_9643_MOESM2_ESM.pdf]

Supplementary Material (Table)

Article title:
Effects of Histidine Protonation and Rotameric States on Virtual Screening of *M. tuberculosis* RmlC

Journal name:
Journal of Computer-Aided Molecular Design

Authors:
Meekyum Olivia Kim[1, *], Sara E. Nichols[1, 2, 3], Yi Wang[4, *, †], J. Andrew McCammon[1, 2, 3, 4]

[1] Department of Chemistry and Biochemistry, University of California San Diego, La Jolla, CA 92093, USA
[2] Department of Pharmacology, University of California San Diego, La Jolla, CA 92093, USA
[3] Center for Theoretical Biological Physics, University of California San Diego, La Jolla, CA 92093, USA
[4] Howard Hughes Medical Institute, University of California San Diego, La Jolla, CA 92093, USA

* To whom correspondence should be addressed:
Meekyum Olivia Kim, mok007@ucsd.edu;
Yi Wang, yiwang@phy.cuhk.edu.hk

† Current address: Department of Physics, The Chinese University of Hong Kong, Shatin, N.T., Hong Kong

	Model	1	2	3	4	5	6	7	8	9	10	11	12	13	14	15	16	17	18	19	20	21	22	23	24	25	26	27	28	29	30	31	32	33	34	35	36
**Model**		HIE62 HIE119	HIE62 FHIE119	HIE62 HID119	HIE62 FHID119	HIE62 HIP119	HIE62 FHIP119	FHIE62 HIE119	FHIE62 FHIE119	FHIE62 HID119	FHIE62 FHID119	FHIE62 HIP119	FHIE62 FHIP119	HID62 HIE119	HID62 FHIE119	HID62 HID119	HID62 FHID119	HID62 HIP119	HID62 FHIP119	FHID62 HIE119	FHID62 FHIE119	FHID62 HID119	FHID62 FHID119	FHID62 HIP119	FHID62 FHIP119	HIP62 HIE119	HIP62 FHIE119	HIP62 HID119	HIP62 FHID119	HIP62 HIP119	HIP62 FHIP119	FHIP62 HIE119	FHIP62 FHIE119	FHIP62 HID119	FHIP62 FHID119	FHIP62 HIP119	FHIP62 FHIP119
**1**	HIE62 HIE119		0.11	**0.00**	0.37	**0.02**	0.10	0.34	0.86	0.11	0.83	**0.01**	0.23	0.13	0.12	0.14	0.14	0.28	0.13	0.09	0.42	0.21	0.12	0.15	0.11	0.13	0.13	0.21	0.82	**0.04**	0.87	0.92	0.71	0.35	0.18	0.65	0.19
**2**	HIE62 FHIE119			**0.01**	**0.02**	**0.00**	**0.04**	0.27	**0.01**	0.19	**0.01**	0.06	0.05	0.12	0.85	0.17	0.35	0.08	0.58	0.47	**0.02**	**0.03**	**0.03**	**0.02**	0.10	0.09	0.81	**0.00**	0.25	**0.03**	0.25	**0.00**	**0.00**	**0.02**	**0.01**	**0.00**	**0.03**
**3**	HIE62 HID119				**0.01**	0.69	**0.01**	**0.00**	**0.01**	**0.01**	**0.01**	**0.01**	**0.00**	**0.01**	**0.01**	**0.01**	**0.01**	**0.03**	**0.01**	**0.01**	**0.00**	**0.01**	**0.01**	**0.01**	**0.01**	**0.00**	**0.01**	**0.01**	0.06	0.97	**0.05**	**0.05**	0.05	**0.01**	**0.03**	**0.01**	**0.01**
**4**	HIE62 FHID119					**0.00**	**0.02**	0.94	**0.00**	**0.02**	**0.02**	0.13	0.72	**0.04**	**0.03**	**0.05**	**0.05**	0.17	**0.04**	**0.01**	0.44	**0.04**	**0.02**	**0.03**	**0.02**	0.20	**0.04**	0.13	0.51	**0.04**	0.53	0.10	**0.00**	0.64	**0.00**	0.08	0.32
**5**	HIE62 HIP119						**0.00**	**0.00**	**0.01**	**0.00**	**0.00**	0.11	**0.00**	**0.01**	**0.01**	**0.01**	**0.01**	0.17	**0.01**	**0.00**	**0.00**	**0.00**	**0.00**	**0.00**	**0.00**	**0.00**	**0.01**	**0.01**	0.13	0.85	0.11	**0.04**	**0.03**	**0.00**	**0.03**	**0.01**	**0.01**
**6**	HIE62 FHIP119							0.24	**0.01**	**0.02**	**0.01**	0.05	**0.05**	**0.00**	**0.00**	**0.00**	**0.00**	0.07	**0.00**	0.26	**0.02**	**0.03**	**0.02**	**0.02**	0.05	0.07	**0.00**	**0.00**	0.23	**0.03**	0.23	**0.00**	**0.00**	**0.02**	**0.01**	**0.01**	**0.03**
**7**	FHIE62 HIE119								0.43	0.28	0.23	0.06	0.97	0.33	0.30	0.33	0.33	**0.01**	0.30	0.24	0.67	0.72	0.34	0.43	0.30	0.67	0.30	0.49	0.23	**0.01**	0.23	0.47	0.21	0.97	**0.00**	0.44	0.75
**8**	FHIE62 FHIE119									**0.01**	0.29	0.31	**0.00**	**0.01**	**0.01**	**0.01**	**0.01**	0.38	**0.01**	**0.00**	0.23	**0.01**	**0.01**	**0.01**	**0.00**	**0.01**	**0.01**	**0.04**	0.78	0.08	0.82	0.94	**0.05**	**0.00**	0.06	0.64	**0.03**
**9**	FHIE62 HID119										**0.01**	0.06	0.06	0.40	0.68	0.44	0.68	0.08	0.98	0.17	**0.03**	**0.04**	**0.04**	**0.02**	0.21	0.09	0.81	**0.00**	0.26	**0.03**	0.26	**0.00**	**0.00**	**0.03**	**0.01**	**0.00**	**0.04**
**10**	FHIE62 FHID119											0.43	**0.01**	**0.02**	**0.01**	**0.02**	**0.02**	0.49	**0.02**	**0.01**	0.08	**0.01**	**0.01**	**0.01**	**0.01**	**0.01**	**0.02**	**0.04**	0.92	0.10	0.97	0.66	0.55	**0.03**	0.09	0.29	**0.03**
**11**	FHIE62 HIP119												0.07	0.07	0.06	0.07	0.07	0.92	0.07	**0.05**	0.12	0.07	0.06	0.07	0.06	**0.05**	0.07	0.10	0.65	0.09	0.59	0.45	0.62	0.13	0.94	0.19	0.07
**12**	FHIE62 FHIP119													0.09	0.07	0.10	0.10	0.14	0.08	**0.04**	0.31	0.27	0.07	0.09	0.06	0.12	0.08	0.23	0.48	**0.03**	0.50	0.15	**0.00**	0.97	**0.00**	**0.00**	0.37
**13**	HID62 HIE119														**0.00**	0.76	0.52	0.09	0.07	0.22	**0.05**	0.08	0.35	0.12	0.73	0.15	**0.03**	**0.00**	0.28	**0.04**	0.28	**0.00**	**0.00**	**0.05**	**0.01**	**0.01**	0.08
**14**	HID62 FHIE119															**0.00**	0.10	0.09	0.47	0.64	**0.04**	0.06	0.12	0.06	0.34	0.11	0.84	**0.00**	0.26	**0.04**	0.26	**0.00**	**0.00**	**0.03**	**0.01**	**0.01**	0.05
**15**	HID62 HID119																0.18	0.10	**0.03**	0.25	0.05	0.09	0.42	0.15	0.79	0.16	**0.00**	**0.00**	0.28	**0.04**	0.28	**0.00**	**0.00**	0.05	**0.01**	**0.01**	0.09
**16**	HID62 FHID119																	0.09	0.12	0.37	**0.05**	0.09	0.36	0.14	0.67	0.16	**0.01**	**0.00**	0.28	**0.04**	0.28	**0.00**	**0.00**	0.05	**0.01**	**0.01**	0.08
**17**	HID62 HIP119																		0.09	0.07	0.15	0.11	0.09	0.10	0.08	0.10	0.09	0.13	0.25	**0.03**	0.17	0.48	0.64	0.19	0.98	0.27	0.11
**18**	HID62 FHIP119																			0.50	**0.04**	0.07	0.22	0.09	0.47	0.13	0.46	**0.00**	0.27	**0.04**	0.27	**0.00**	**0.00**	**0.04**	**0.01**	**0.01**	0.07
**19**	FHID62 HIE119																				**0.01**	**0.02**	**0.00**	**0.00**	**0.00**	0.05	0.64	**0.00**	0.23	**0.03**	0.23	**0.00**	**0.00**	**0.02**	**0.00**	**0.00**	**0.02**
**20**	FHID62 FHIE119																					**0.02**	**0.03**	**0.03**	**0.02**	**0.04**	**0.04**	0.12	0.54	**0.04**	0.57	0.34	**0.03**	0.49	**0.00**	0.09	0.06
**21**	FHID62 HID119																						**0.04**	0.06	**0.03**	0.73	0.07	0.27	0.40	**0.03**	0.41	**0.02**	**0.00**	0.34	**0.00**	**0.00**	0.82
**22**	FHID62 FHID119																							**0.02**	0.18	0.12	0.19	0.09	0.28	**0.03**	0.29	**0.00**	**0.00**	**0.03**	**0.01**	**0.00**	**0.05**
**23**	FHID62 HIP119																								**0.01**	0.20	0.08	0.94	0.32	**0.04**	0.32	**0.00**	**0.00**	**0.04**	**0.01**	**0.00**	0.07
**24**	FHID62 FHIP119																									0.10	0.43	**0.02**	0.26	**0.03**	0.26	**0.00**	**0.00**	**0.02**	**0.00**	**0.00**	**0.03**
**25**	HIP62 HIE119																										0.13	0.40	0.39	**0.03**	0.40	**0.03**	**0.00**	0.37	**0.00**	**0.00**	0.25
**26**	HIP62 FHIE119																											**0.00**	0.27	**0.04**	0.27	**0.00**	**0.00**	**0.04**	**0.01**	**0.01**	0.06
**27**	HIP62 HID119																												0.35	0.05	0.35	**0.00**	**0.00**	0.16	**0.02**	**0.04**	0.26
**28**	HIP62 FHID119																													**0.01**	0.30	0.82	0.99	0.51	0.58	0.69	0.41
**29**	HIP62 HIP119																														**0.01**	0.15	0.18	**0.05**	0.15	0.07	**0.04**
**30**	HIP62 FHIP119																															0.85	0.94	0.53	0.50	0.73	0.43
**31**	FHIP62 HIE119																																0.18	0.07	0.20	0.70	**0.04**
**32**	FHIP62 FHIE119																																	**0.00**	0.33	0.13	**0.00**
**33**	FHIP62 HID119																																		**0.01**	0.12	0.55
**34**	FHIP62 FHID119																																			**0.03**	**0.00**
**35**	FHIP62 HIP119																																				**0.00**
**36**	FHIP62 FHIP119																																				

Supplementary Table 1 *p*-Values for AUC of 36 receptor models, displayed with protonation and rotameric states of two histidines in each model. The letter F in protonation and rotameric state name represents a flipped state. Receptors with *p*-Values less than 0.05 (boldfaced) differ in their ability to distinguish the actives and decoys in VS with statistical significance compared to others.
